# Prediction of Zonisamide Concentration in Pediatric Patients With Epilepsy: A Machine Learning Approach

**DOI:** 10.1002/cns.70996

**Published:** 2026-06-22

**Authors:** Li Fashuang, Ma Mingbiao, Li Na, Li Huiying, Ren Danyang, Tu Caixia, Li Linbo, Zhang Lilin

**Affiliations:** ^1^ Department of Pharmacy Kunming Children's Hospital Kunming China; ^2^ Laboratory Department Kunming Children's Hospital Kunming China; ^3^ Department of Pharmacy Yunnan Provincial Hospital of Infectious Disease Kunming China

**Keywords:** children, epilepsy, machine learning, Zonisamide

## Abstract

**Objective:**

To construct and validate prediction models for zonisamide (ZNS) concentration in pediatric patients with epilepsy based on 12 machine learning algorithms, to screen for the optimal algorithm, and to provide a scientific basis for the formulation of individualized ZNS dosing regimens in children.

**Methods:**

Clinical data of patients who underwent ZNS therapeutic drug monitoring at Kunming Children's Hospital from May 2022 to January 2026 were retrospectively collected and randomly divided into a training set and a test set at a ratio of 7:3. Key predictive variables were determined through a multi‐stage feature screening strategy (covering correlation analysis, collinearity diagnosis, univariate analysis, Lasso regression, and random forest algorithm). Based on the selected variables, 12 machine learning regression models were constructed to predict ZNS concentration, and grid search combined with 5‐fold cross‐validation was employed for parameter optimization and performance evaluation. The coefficient of determination (*R* ^2^), mean squared error (MSE), root mean squared error (RMSE), and mean absolute error (MAE) were used as primary evaluation metrics. Finally, the SHAP method was adopted to interpret the feature contribution and decision logic of the optimal model.

**Results:**

The modeling cohort of this study enrolled 532 pediatric patients who received zonisamide treatment at the Department of Neurology, Kunming Children's Hospital from May 2022 to May 2024. These patients were randomly divided into a training set (375 cases) and an internal validation set (157 cases) at a ratio of 7:3. Additionally, 436 pediatric patients from the same department of the same hospital from June 2024 to January 2026 were included as an external validation cohort. Comparisons of general clinical data, laboratory indicators, and medication‐related data among the groups showed no statistically significant differences (all *p* > 0.05), indicating that the baseline data were balanced and comparable. The median (interquartile range) ZNS concentrations in the training set, internal validation set, and external validation cohort were 9.65 (8.11, 11.98) μg/mL, 9.93 (8.23, 12.06) μg/mL, and 10.77 (7.67, 13.69) μg/mL, respectively. Through multi‐stage feature screening, gender, dosage, age, red blood cell count, concomitant medication status, total protein, uric acid, and platelets were identified as key factors influencing ZNS concentration. Among the 12 constructed machine learning models, the Random Forest (RF) algorithm demonstrated the optimal performance: in the training set, *R* ^2^ was 0.97%, RMSE was 0.83%, MAE was 0.57%, and Err20 was 7.40%; in the internal validation set, *R* ^*2*^ was 0.78%, RMSE was 1.99%, MAE was 1.48%, and Err20 was 31.20%; and in the external validation set, *R* ^2^ was 0.89%, RMSE was 1.31%, MAE was 0.86%, and Err20 was 16.50%. SHAP method combined with representative decision tree analysis revealed that dosage and gender were the primary factors influencing ZNS concentration, followed by total protein and uric acid; among them, dosage showed a positive contribution, gender exhibited a bidirectional effect, and laboratory indicators mostly showed nonlinear associations. Decision tree analysis indicated that the model used gender as the primary splitting feature and incorporated multi‐indicator interactions; its decision logic aligned with pharmacokinetic theory, providing strong support for the interpretability of the model's clinical application.

**Conclusion:**

This study successfully constructed and validated a ZNS concentration prediction model for pediatric patients with epilepsy based on the random forest algorithm. The model demonstrated high precision, strong stability, and good generalization ability. Gender, dose, uric acid, and total protein are core variables influencing ZNS concentration. The findings can provide a reference for the formulation of individualized ZNS dosing regimens in children.

## Graphical abstract

The graphical abstract illustrates a study on the prediction of plasma zonisamide concentrations in pediatric patients with epilepsy. Utilizing clinical indicators, the study employed a multi‐stage feature screening process to construct and compare 12 machine learning models. Ultimately, the random forest model was identified as optimal, achieving *R* ^2^ values of 0.965 and 0.894 on the training and validation sets, respectively. Model interpretation via SHAP analysis revealed that dosage and gender were critical determinants of plasma concentration. This study aims to facilitate safer and more precise zonisamide dosing regimens for pediatric patients.

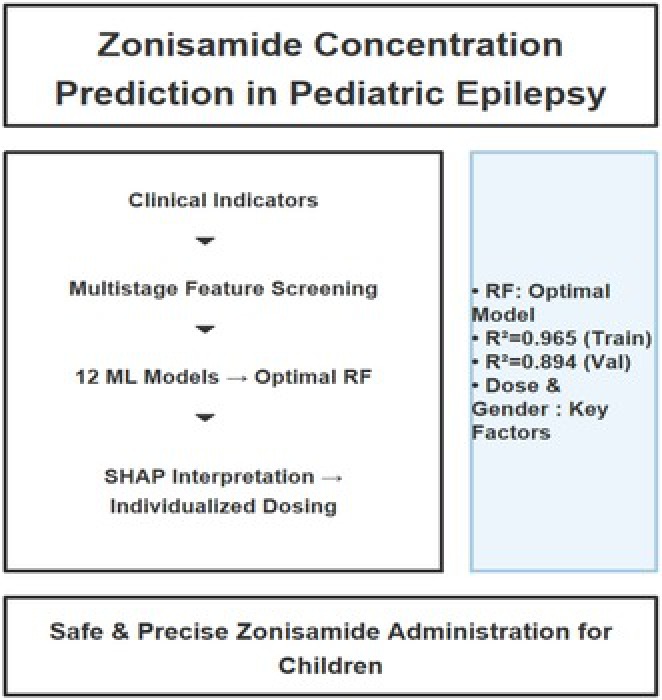

## 1 Introduction

Epilepsy is one of the most common chronic neurological disorders in childhood, and long‐term pharmacotherapy serves as the primary modality for controlling epileptic seizures [1, 2]. ZNS, a benzisoxazole derivative characterized by a unique chemical structure and multiple mechanisms of action, has been widely employed as monotherapy or adjunctive therapy for focal epilepsy in the pediatric population [3]. However, owing to the fact that children are in the developmental stage, their physiological functions (such as hepatic and renal metabolic functions and body fluid distribution) exhibit significant age‐related variations. Furthermore, enzyme induction or inhibition resulting from the concomitant use of antiepileptic drugs leads to significant inter‐individual variability and nonlinear characteristics in the pharmacokinetic (PK) behavior of ZNS in children [4, 5]. Additionally, the in vivo process of ZNS is influenced by various factors, including age, body weight, hepatic and renal function, and concomitant medications. Excessively high concentrations are prone to induce adverse reactions such as somnolence, dizziness, and ataxia, whereas subtherapeutic concentrations fail to effectively control epileptic seizures [6]. A case series study indicated that the effective concentration range of ZNS is 10.2–31.9 μg/mL [7], a range within which the frequency of epileptic seizures can be effectively reduced. In that study, 6 patients discontinued treatment due to poor efficacy, suggesting that some patients may possess pharmacodynamic resistance or pharmacokinetic differences that result in blood drug concentrations failing to reach the therapeutic range. When concentrations exceed the upper limit of the therapeutic window, the risk of adverse reactions increases significantly. Therefore, it is clinically recommended to assess drug exposure via Therapeutic Drug Monitoring (TDM) to ensure that concentrations remain within the therapeutic window, thereby balancing efficacy and safety. If patient concentrations fall outside the therapeutic range, dosage adjustments or alternative treatment regimens should be implemented promptly.

However, TDM only provides single‐point concentration measurements and lacks predictive capability, making it difficult to optimize therapeutic regimens for complex patients with substantial pharmacokinetic variability [8]. To achieve prospective individualized dosing, it is necessary to integrate advanced computational models to accurately predict concentrations. Traditional population pharmacokinetic models are constrained by pre‐defined mathematical structures and have limited capacity to capture complex nonlinear relationships and variable interactions [9]. In recent years, machine learning techniques have demonstrated advantages over traditional statistical methods in the field of pharmacokinetic prediction due to their powerful capabilities in feature extraction, nonlinear fitting, and complex data processing [10, 11, 12, 13]. Previous studies have confirmed their valuable application in predicting the concentrations of other antiepileptic drugs in patients with epilepsy; however, a mature prediction system specifically for ZNS has not yet been established.

Current research on machine learning prediction models for ZNS remains limited. Although machine learning models possess theoretical superiority, applied research in the field of ZNS is relatively scarce, largely remaining at the stage of pharmacokinetic characterization or general algorithm discussion and lacking large‐scale, prospective clinical validation data. In the existing literature, while there are successful cases of machine learning prediction for other drugs (such as linezolid and antineoplastic agents), mature machine learning prediction models specifically targeting ZNS have not been widely reported [14]. Based on this, this study retrospectively collected clinical data and concentration data from pediatric epilepsy patients treated with ZNS at Kunming Children's Hospital. Prediction models based on 12 machine learning algorithms were constructed; model performance was evaluated using multidimensional metrics to select the optimal algorithm, and the SHAP method was employed to interpret model features, aiming to provide a scientific and precise theoretical basis and practical tool for the formulation of ZNS dosing regimens in children.

## 2 Materials and Methods

### 2.1 Study Participants

**FIGURE 1:**
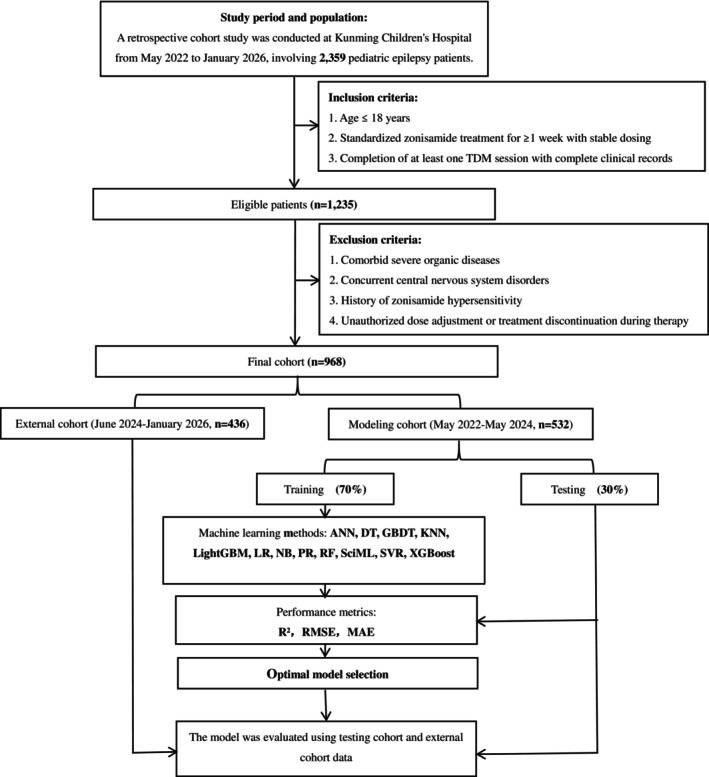
Study on individualized dosing prediction model of zonisamide in children with epilepsy: Flowchart of patient screening and machine learning model construction.

This study enrolled 532 pediatric patients who received ZNS treatment at the Department of Neurology, Kunming Children's Hospital, between May 2022 and May 2024, constituting the modeling cohort. Additionally, 436 pediatric patients treated between June 2024 and January 2026 were enrolled as the external validation cohort (the patient screening process is detailed in Figure 1). Included participants were children and adolescents aged ≤ 18 years who met the diagnostic criteria for epilepsy outlined in the Clinical Practice Guidelines: Epilepsy Volume (2022 Edition) [15] and presented with recurrent seizures requiring ZNS therapy. The specific inclusion criteria were as follows: (1) a confirmed diagnosis of epilepsy based on clinical history, neurological examination, and necessary electroencephalogram (EEG) or neuroimaging findings, excluding febrile seizures, reflex seizures, or other non‐epileptic paroxysmal events; (2) age ≤ 18 years; (3) treatment with ZNS monotherapy or adjunctive therapy for ≥ 2 weeks with a stable dosage and good adherence; (4) completion of at least one TDM session with complete clinical data; and (5) unique patient records to ensure no duplicate entries (each record contained a distinct ZNS concentration measurement). The exclusion criteria included: (1) comorbid severe hepatic or renal failure, heart failure, or hematological disorders; (2) coexisting central nervous system diseases (e.g., encephalitis, meningitis, or brain tumors); (3) history of allergy or intolerance to ZNS; and (4) unauthorized dose adjustments, treatment interruption, or concomitant use of non‐antiepileptic drugs known to affect ZNS metabolism.

### 2.2 Ethics and Informed Consent Statement

All clinical data in this study underwent rigorous de‐identification. Sensitive information capable of identifying individuals, including patient names, identification numbers, home addresses, and contact details, was completely removed; only anonymized clinical data were retained for statistical analysis. No written consent has been obtained from the patients as there is no patient‐identifiable data included.

### 2.3 Zonisamide Dosage Regimen

In accordance with the center's routine pediatric dosing strategy and the manufacturer's instructions, the ZNS regimen was initiated at 1–2 mg/(kg·day), administered orally in 1–2 divided doses. The dosage was titrated every 1–2 weeks based on seizure control and tolerability, with increments not exceeding 2 mg/(kg·day). The routine maintenance dose ranged from 2 to 4 mg/(kg·day), which could be increased to a maximum of 8 mg/(kg·day) for refractory epilepsy or adjunctive therapy. Dose adjustments were made for patients concomitantly taking hepatic enzyme‐inducing antiepileptic drugs. The weight‐standardized daily dose (mg/(kg·day), calculated as total daily dose divided by measured body weight) was used as the core dosing variable to ensure comparability. Steady‐state concentration was defined as maintaining a once‐daily regimen for ≥ 7 days or a twice‐daily regimen for ≥ 5 days. Samples were collected in the morning, 30 min prior to the next dose, under fasting conditions to determine steady‐state trough concentrations. Dosage stability was defined as no adjustments for ≥ 2 consecutive weeks and no self‐reported missed doses. Adherence was monitored via patient/family diaries, requiring a missed dose rate of < 5% and no significant treatment interruptions to provide a standardized reference for TDM and model construction.

### 2.4 Therapeutic Drug Monitoring

ZNS trough concentrations were measured after achieving steady state. Venous samples (2 mL) were drawn 30 min prior to the morning dose. ZNS concentrations were determined using ultra‐performance liquid chromatography (UPLC). Samples were pretreated with 7% perchloric acid solution, and 20 μL of the supernatant was injected for analysis. Chromatographic separation was performed on an ACQUITY UPLC Peptide BEH C18 column (2.1 mm × 50 mm, 1.7 μm) with a mobile phase of 0.1% formic acid–acetonitrile (85:15) at a flow rate of 0.20 mL min^−1^. The column temperature was maintained at 40°C, and detection was performed at 265 nm. The method demonstrated good linearity within the range of 0.50–50.00 μg mL^−1^ (*r* ^2^ > 0.9996). The extraction recovery rate ranged from 96.90% to 103.70%. Intra‐day and inter‐day precision were < 7.63% and < 8.22%, respectively, and the relative standard deviation (RSD) for stability was < 14.85%.

### 2.5 Data Collection

Clinical data were retrieved from the hospital's electronic medical records and laboratory information systems. The collected variables included: (1) Demographic characteristics: age, sex, body weight; (2) ZNS administration data: dosage, treatment duration, concomitant medications; (3) Laboratory findings: white blood cell count (WBC), red blood cell count (RBC), hemoglobin (HGB), hematocrit (HCT), platelet count (PLT), alanine aminotransferase (ALT), aspartate aminotransferase (AST), total bilirubin (TBiL), total protein (TP), albumin (ALB), serum creatinine (SCr), glomerular filtration rate (GFR), blood urea nitrogen (BUN), uric acid (UA), fasting blood glucose (FBG), and vitamin D (VitD); (4) ZNS concentration.

All clinical and laboratory data were collected at baseline, defined as the period after dose stabilization and prior to TDM sampling, rather than synchronously on the day of sampling. Sex was recorded as a fixed demographic variable. For dynamic clinical and laboratory indicators, the most recent results obtained prior to TDM sampling were used as baseline values. This approach ensures the model's utility for prospective prediction of steady‐state concentrations and avoids the limited added value associated with using synchronous sampling data.

### 2.6 Data Preprocessing

All preprocessing parameters in this study were derived exclusively from the training set and subsequently applied uniformly to the test set and the external validation cohort. The specific preprocessing steps were as follows: (1) Dataset partitioning: The modeling cohort was randomly divided into a training set (*n* = 375) and a test set (*n* = 157) at a ratio of 7:3. (2) Missing value imputation: The mean of continuous variables and the mode of categorical variables were calculated based on the training set; subsequently, these values were utilized to impute missing data in the training set, test set, and external validation set. (3) Outlier handling: Based on the statistics of the training set, the interquartile range (IQR) method was employed to determine the thresholds for outlier detection. Subsequently, outliers in the test set and external validation set were managed via winsorization using these thresholds. (4) Categorical variable encoding: Based on the distribution within the training set, dummy encoding was performed on binary variables such as gender and concomitant medication. Specifically, gender was encoded as male = 1 and female = 2, while concomitant medication was encoded as no = 0 and yes = 1; this unified encoding scheme was applied to all validation sets. (5) Continuous variable standardization: Based on the mean and standard deviation derived from the training set, the Z‐score standardization method was adopted to standardize all continuous variables. These standardization parameters were synchronized across all validation sets to eliminate dimensional and scale differences among variables.

### 2.7 Feature Selection

All feature screening procedures in this study were conducted exclusively within the training cohort to prevent data leakage. The specific implementation steps were as follows: (1) Diagnosis of correlation and multicollinearity: Spearman correlation analysis was performed on the training set variables, and the Variance Inflation Factor (VIF) was calculated for each variable. Variables with a VIF ≥ 10.00 were excluded to eliminate the interference of multicollinearity with the model, while those with a VIF < 10.00 were retained for subsequent analysis. (2) Univariate screening: Univariate linear regression analysis was conducted on the variables remaining after collinearity screening. Variables exhibiting a statistically significant association with ZNS concentration in the training set (*p* < 0.05) were selected for further analysis. (3) Preliminary screening based on Lasso regression: Variables demonstrating statistical significance in the univariate analysis were incorporated into a Lasso regression model. The optimal regularization parameter (*λ*) was determined via 10‐fold cross‐validation combined with grid search on the training set. Variables with non‐zero regression coefficients and absolute coefficient values ≥ 0.05 were retained to exclude weakly correlated variables, thereby constructing a candidate feature set. (4) Multi‐algorithm joint verification and feature determination: The boruta algorithm was employed to evaluate the validity of the candidate features screened by lasso regression; only variables with importance significantly higher than shadow features and marked as “Confirmed” were retained. Concurrently, the stability of feature selection was assessed based on variable selection frequency through 500 bootstrap resampling iterations. Finally, the core feature set for modeling was determined by integrating Lasso regression coefficient constraints, Boruta feature importance verification, and Bootstrap stability test results. Upon completion of feature screening, the fixed feature subset identified in the training set was directly applied to the test set and external validation set; no secondary screening or parameter adjustment was performed on the external cohort data.

This study employed a quantitative multi‐layer inclusion criteria, integrating lasso regression results with feature importance analysis to complete feature screening. Variables lacking practical predictive value were eliminated via the lasso regression coefficient threshold. The boruta algorithm was utilized to distinguish effective features from random noise and quantify variable predictive efficacy. Furthermore, multiple resampling validations were conducted to assess the inclusion stability of features across different subsamples, ensuring that the final features possessed stable and reproducible predictive performance. In this study, variables that did not pass the univariate test were directly excluded; the final modeling variables were all derived from the qualified candidate set following the stepwise screening. This standardized and rigorous stepwise screening process provided reliable variable support for the construction of the ZNS concentration prediction model.

### 2.8 Model Construction and Performance Evaluation

Twelve commonly used machine learning and statistical regression algorithms were employed to construct prediction models for ZNS concentration. These algorithms included Artificial Neural Network (ANN), Decision Tree (DT), Gradient Boosting Decision Tree (GBDT), K‐Nearest Neighbor (KNN), Poisson Regression (PR), Light Gradient‐Boosting Machine (LightGBM), Linear Regression (LR), Negative Binomial Regression (NB), Poisson Regression (PR), RF, Support Vector Regression (SVR), and eXtreme Gradient Boosting (XGBoost). Predictive analyses were conducted using these models to identify the optimal prediction model. Model fitting was performed on the training set of the modeling cohort. Grid search combined with 10‐fold cross‐validation was utilized for hyperparameter optimization and internal validation to determine the optimal architecture for each model. The performance of all models was comprehensively evaluated and compared on an independent internal test set to select the model with the superior predictive effect.

### 2.9 Model Performance Metrics and Interpretation of the Optimal Model

Three regression evaluation metrics were selected: (1) *R* ^2^: Ranging from 0.00 to 1.00, values closer to 1.00 indicate stronger explanatory power regarding data variance and better predictive performance. (2) RMSE: This measures the root mean square deviation between predicted and actual values; smaller values indicate higher prediction accuracy. (3) MAE: This reflects the mean absolute deviation between predicted and actual values; smaller values represent better model stability. Based on these metrics, performance comparisons of all models were conducted using 10‐fold cross‐validation results from the training set to ensure the stability and reliability of the evaluation. Further validation was performed on the independent internal test set, and the optimal model was selected by synthesizing results from both phases. Finally, the Shapley Additive Explanations (SHAP) method was applied to interpret the optimal model, clarifying the magnitude and direction of influence of key features on the predicted ZNS concentration.

### 2.10 Statistical Analysis

Statistical analysis was performed using R software (version 4.5.2). Continuous variables not following a normal distribution were expressed as median (interquartile range) [M (Q1, Q3)], and between‐group comparisons were performed using the Mann–Whitney *U* test. Categorical variables were expressed as count (percentage) [*n* (%)], and between‐group comparisons were conducted using the chi‐square (*χ* ^2^) test. A *p‐*value < 0.05 was considered statistically significant.

## 3 Results

### 3.1 General Information

A total of 968 pediatric patients with epilepsy were enrolled in this study, and 968 steady‐state trough concentration monitoring data points of zonisamide were obtained (each patient contributed only one record, with no repeated measurements). The study sample was divided into a modeling cohort (532 cases) and an external validation cohort (436 cases). Specifically, the modeling cohort was randomly divided into a training set (375 cases) and a test set (157 cases) at a ratio of 7:3.

In the modeling cohort, there were 313 males (58.83%) and 219 females (41.17%); the age ranged from 1.08 to 17.50 years, and the body weight ranged from 8.19 to 73.10 kg. Comparison of baseline characteristics between the training set and the test set showed: in the training set, there were 219 males (58.40%) and 156 females (41.60%), with an age range of 1.33–17.63 years and a body weight range of 6.78–69.81 kg; in the test set, there were 94 males (59.87%) and 63 females (40.13%), with an age range of 1.42–15.83 years and a body weight range of 9.00–83.33 kg. There were no statistically significant differences in baseline characteristics between the two groups (*p* > 0.05), indicating comparability. The external validation cohort comprised 436 cases, including 261 males (59.86%) and 175 females (41.55%); the age ranged from 1.13 to 16.77 years, and the body weight ranged from 7.39 to 70.21 kg. There was no statistically significant difference in baseline characteristics between the external validation cohort and the modeling cohort (*p* > 0.05), similarly indicating comparability (Table 1).

**TABLE 1:** Comparison of baseline characteristics in children.

Variable	Training cohort (*n* = 375)	Testing cohort (*n* = 157)	*p*	Modeling cohort (*n* = 532)	External validation cohort (*n* = 436)	*p*
Gender (male/female)	219/156	94/63	0.476	313/219	261/175	0.796
Age (years)	8.67 (6.83, 10.96)	8.58 (7.08, 10.42)	0.978	8.67 (6.83, 10.67)	8.42 (6.75, 10.17)	0.103
Weight (kg)	31.00 (22.50, 40.60)	30.70 (22.50, 39.10)	0.565	31.00 (22.50, 40.50)	29.60 (21.90, 38.70)	0.079
Concomitant (0/1)	304/71	132/25	0.402	436/96	354/82	0.825
Dose (mg/(kg・day))	2.43 (2.06, 2.72)	2.43 (2.18, 2.68)	0.579	2.43 (2.10, 2.71)	2.44 (2.17, 2.71)	0.263
Duration (months)	8.60 (5.00, 15.65)	11.30 (5.20, 16.50)	0.199	9.10 (5.00, 15.73)	8.60 (5.00, 5.30)	0.392
WBC (10^9^/L)	6.43 (5.63, 7.65)	6.45 (5.48, 7.56)	0.670	6.43 (5.61, 7.60)	6.48 (5.61, 7.74)	0.599
RBC (10^12^/L)	4.91 (4.61, 5.21)	4.92 (4.61, 5.16)	0.690	4.92 (4.61, 5.19)	4.92 (4.61, 5.21)	0.949
HGB (g/L)	139.00 (133.00, 145.50)	138.00 (133.00, 145.00)	0.691	138.00 (133.00, 145.25)	138.00 (132.00, 146.00)	0.946
PLT (10^9^/L)	298.00 (262.50, 358.50)	304.00 (270.00, 356.00)	0.992	299.50 (264.50, 358.25)	293.00 (268.75, 348.75)	0.541
HCT (%)	40.50 (38.20, 42.55)	40.50 (39.10, 42.60)	0.222	40.50 (38.50, 42.60)	40.40 (38.28, 42.70)	0.912
ALT (U/L)	15.00 (13.00, 20.00)	16.00 (13.00, 20.00)	0.927	16.00 (13.00, 20.00)	15.00 (12.00, 20.00)	0.584
AST (U/L)	25.00 (20.00, 29.00)	23.00 (20.00, 27.00)	0.211	24.00 (20.00, 28.00)	25.00 (21.00, 29.00)	0.060
TBIL (μmol/L)	10.80 (8.75, 13.45)	11.30 (8.60, 13.40)	0.702	10.90 (8.70, 13.43)	10.90 (8.60, 13.30)	0.759
TP (g/L)	72.90 (68.80, 76.40)	72.80 (68.30, 75.50)	0.303	72.80 (68.68, 76.13)	72.20 (68.00, 76.10)	0.234
ALB (g/L)	43.20 (41.25, 45.70)	43.30 (41.50, 45.60)	0.822	43.20 (41.30, 45.63)	43.15 (41.10, 45.70)	0.704
SCr (μmol/L)	47.00 (39.00, 56.00)	47.00 (39.00, 55.00)	0.550	47.00 (39.00, 56.00)	46.00 (38.00, 54.00)	0.100
GFR (mL/(min·1.73m^2^))	100.39 (83.82, 130.09)	99.35 (81.86, 127.19)	0.796	100.05 (83.40, 128.75)	98.90 (81.80, 127.75)	0.429
BUN (mmol/L)	4.88 (4.15, 5.75)	4.88 (4.13, 5.51)	0.999	4.88 (4.13, 5.67)	4.86 (4.13, 5.63)	0.683
UA (μmol/L)	288.80 (254.00, 356.50)	286.50 (248.90, 334.60)	0.406	287.90 (249.78, 353.83)	283.60 (243.53, 356.83)	0.451
FBG (mmol/L)	5.28 (4.86, 5.64)	5.21 (4.90, 5.50)	0.835	5.26 (4.89, 5.62)	5.28 (4.87, 5.59)	0.999
VitD (nmol/L)	61.50 (51.07, 71.52)	63.89 (54.73, 72.56)	0.124	62.30 (51.90, 71.95)	62.51 (53.67, 72.52)	0.505
Concentration (μg/mL)	9.65 (8.11, 11.98)	9.93 (8.23, 12.06)	0.865	9.83 (8.25, 12.01)	10.77 (7.67–13.69)	0.238

*Note:* Continuous variables are expressed as median (interquartile range, IQR), and categorical variables are expressed as the number of cases. Comparisons between groups were performed using the Mann–Whitney *U* test (for continuous variables) and the *χ* ^2^ test (for categorical variables), with *p* < 0.05 considered statistically significant. All clinical and laboratory covariates in this study were baseline data collected prior to TDM sampling, aiming for the prospective prediction of zonisamide steady‐state concentration, rather than data measured concurrently with blood sampling; the consistency of baseline data ensured the comparability of the training set, test set, and external validation set, laying the foundation for evaluating the generalization ability of the model.

Abbreviations: ALB, albumin; ALT, alanine aminotransferase; AST, aspartate aminotransferase; BUN, blood urea nitrogen; Cr, creatinine; DD, weight‐standardized dose; FBG, fasting blood glucose; GFR, glomerular filtration rate; HCT, hematocrit; HGB, hemoglobin; PLT, platelet; RBC, red blood cell; TBIL, total bilirubin; TP, total protein; UA, uric acid; VitD, vitamin D; WBC, white blood cell.

### 3.2 Screening of Key Factors Influencing Zonisamide Concentration in Children With Epilepsy and Stability Verification Results

#### 3.2.1 Correlation and Multicollinearity Analysis of Clinical Variables

**FIGURE 2:**
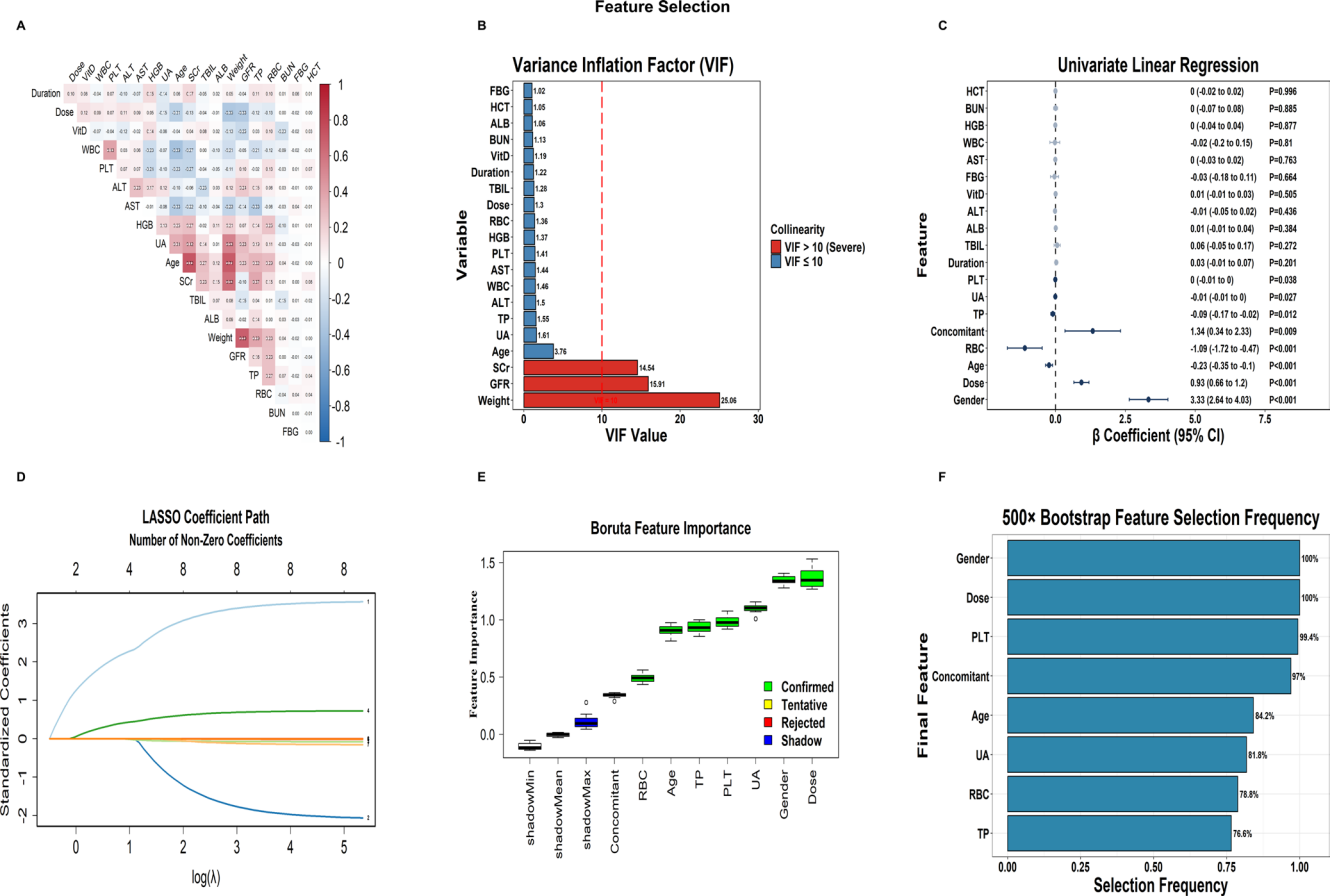
Screening of key factors influencing zonisamide concentration in children with epilepsy and stability verification results. (A) Correlation matrix heatmap of clinical characteristic variables in children with epilepsy. The heatmap displays pairwise Spearman correlation coefficients among demographic, clinical, and laboratory parameters. Red indicates a positive correlation, while blue indicates a negative correlation; color intensity is proportional to the magnitude of the correlation (range: −1 to 1). (B) Histogram of variance inflation factors (VIF) for clinical characteristic variables to assess multicollinearity. The red dashed line represents the threshold of VIF = 10; variables with VIF > 10 suggest significant multicollinearity. (C) Univariate linear regression forest plot of clinical characteristic variables in children with epilepsy. The plot illustrates the regression coefficients (*β*‐coefficient) and their 95% confidence intervals (95% CI) for each variable. Blue dots represent the estimated regression coefficients, and horizontal lines denote the 95% CI. Statistical significance in univariate analysis (*p* < 0.05) is indicated when the confidence interval does not cross the null line (*β* = 0, represented by the dashed line). (D) Lasso regression coefficient path diagram. The trajectory shows the change in standardized regression coefficients for each feature as a function of the negative logarithm of the regularization parameter (−log(*λ*)). The *x*‐axis represents −log(*λ*), where larger values indicate weaker regularization constraints; the *y*‐axis represents the standardized regression coefficient values, with the zero line as the baseline. As regularization intensity decreases, regression coefficients gradually deviate from zero and converge to their final model values. The numbers at the top indicate the count of non‐zero coefficients. (E) Box plot of feature importance using the Boruta algorithm. The y‐axis represents the feature importance score, and the x‐axis lists the variable names. The box plots display the distribution of feature importance for each variable; green boxes represent variables labeled as “Confirmed,” indicating that their importance is significantly higher than shadow features and that they make a genuine contribution to model prediction performance. (F) Bar chart of feature selection frequencies for each variable under 500 Bootstrap resampling iterations. The *y*‐axis lists variable names, and the *x*‐axis represents the selection frequency during Bootstrap resampling (range: 0–1); higher frequencies suggest stronger predictive stability of the variables. ALB, albumin; ALT, alanine aminotransferase; AST, aspartate aminotransferase; BUN, blood urea nitrogen; FBG, fasting blood glucose; GFR, glomerular filtration rate; HCT, hematocrit; HGB: hemoglobin; PLT, platelet; RBC, red blood cell; SCr, serum creatinine; TBIL, total bilirubin; TP, total protein; UA, uric acid; VitD, vitamin D; WBC, white blood cell.

First, Spearman's correlation coefficient was used to analyze the correlation of clinical variables. The results showed that body weight was strongly positively correlated with age (*r* = 0.80, |*r*| > 0.80); among renal function indicators, SCr was strongly negatively correlated with GFR (*r* < −0.80); whereas the absolute values of correlation coefficients between indicators such as TBIL, TP, UA, FBG, and VitD and most variables were < 0.30, indicating weak correlations (Figure 2A).

Multicollinearity diagnosis results showed that the VIF of each variable ranged from 1.02 to 25.06. Among them, the VIF values for body weight (VIF = 25.06), GFR (VIF = 15.91), and SCr (VIF = 14.54) were all greater than 10.00, suggesting significant multicollinearity. To eliminate the interference of multicollinearity on subsequent modeling, this study excluded variables with VIF ≥ 10.00 (body weight, GFR, SCr), and the remaining variables were included in the subsequent analysis (Figure 2B).

#### 3.2.2 Univariate Linear Regression Analysis

Univariate linear regression analysis was performed on the remaining indicators after excluding multicollinear variables. The results showed that Gender, Dose, Age, RBC, Concomitant, TP, UA, and PLT were all significantly correlated with ZNS concentration in pediatric patients with epilepsy (*p* < 0.05) (Figure 2C).

#### 3.2.3 Lasso Regression Feature Screening

The 8 variables with statistical significance in the univariate analysis were included in the Lasso regression model, and the optimal regularization parameter *λ* (*λ* = 0.45) was determined via grid search combined with 10‐fold cross‐validation. Under the optimal *λ* condition, no variables had their coefficients compressed to 0, suggesting that the above variables all possessed certain predictive value and could serve as candidate features for the next step of screening (Figure 2D).

#### 3.2.4 Boruta Feature Importance Evaluation

To further enhance the stability and rationality of feature selection, the Boruta algorithm was employed to evaluate feature importance using the candidate variables retained by Lasso regression as input. The results showed that the importance of all included candidate variables (Concomitant, RBC, Age, TP, PLT, UA, Gender, Dose) was significantly higher than the maximum value of shadow features, and all were marked as “confirmed,” suggesting that these variables all made real contributions to the predictive performance of the model. Importance ranking showed that Dose had the highest importance score, followed by Gender, both of which were significantly higher than other variables; the importance of RBC and Concomitant was relatively lower but still significantly higher than the shadow feature level, and thus they were not excluded (Figure 2E).

#### 3.2.5 Bootstrap Resampling Validation of Feature Stability

To further verify the stability of feature selection, this study used 500 bootstrap resampling iterations to evaluate the inclusion frequency of the above variables. The results showed that the inclusion frequencies of each variable ranged from 76.60% to 100.00%; specifically, the inclusion frequencies for Gender and Dose were 100.00%, PLT was 99.40%, Concomitant was 97.00%, Age was 84.20%, UA was 81.80%, RBC was 78.80%, and TP was 76.60%, indicating good overall stability (Figure 2F). Ultimately, the above 8 variables were determined as important predictive variables for the subsequent construction of the ZNS concentration prediction model.

### 3.3 Model Performance Validation and Determination of the Optimal Model

**FIGURE 3:**
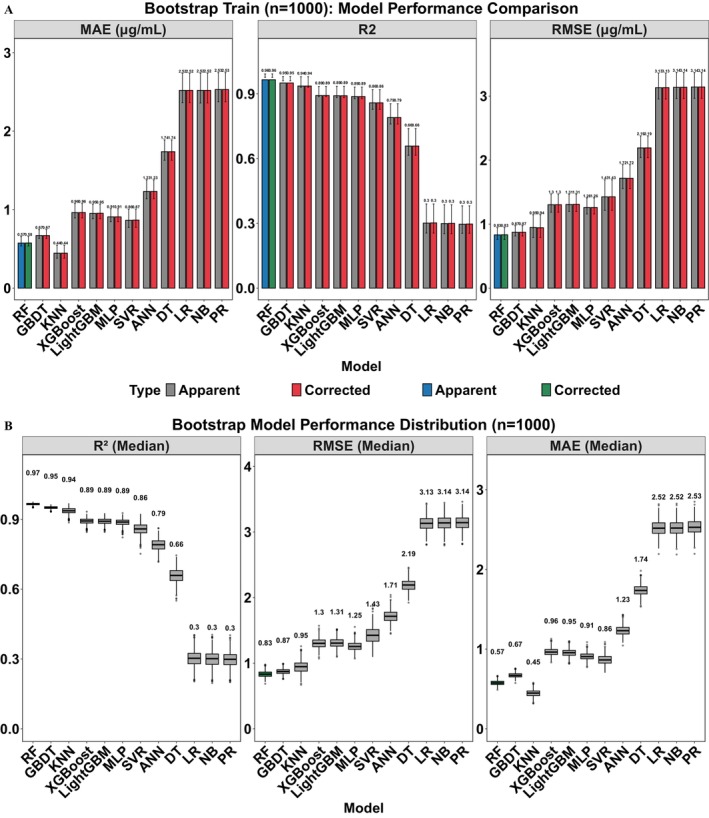
Model performance evaluation results. (A) Bar charts depicting the mean ± standard deviation of performance metrics for each model. The figure illustrates the coefficient of determination (*R* ^2^), root mean square error (RMSE), and mean absolute error (MAE). Gray bars represent apparent performance, while red bars indicate Bootstrap bias‐corrected performance, visually reflecting the degree of model overfitting and performance bias. (B) Box plots illustrating the distribution of performance metrics. The distribution ranges and medians of *R* ^2^, RMSE, and MAE are displayed based on 1000 resampling iterations to evaluate the predictive stability of the models across different data subsets. Evaluation metrics: *R* ^2^ denotes the coefficient of determination, where a value closer to 1 indicates stronger explanatory power regarding the outcome variable; RMSE and MAE are error metrics, where lower values signify higher predictive accuracy. ANN, Artificial Neural Network; DT, Decision Tree; GBDT, Gradient Boosting Decision Tree; KNN, K‐Nearest Neighbors; LightGBM, Light Gradient Boosting Machine; LR, Linear Regression; NB, Negative Binomial Regression; PR, Poisson Regression; RF, Random Forest; SciML, Scientific Machine Learning; SVR, Support Vector Regression; XGBoost, Extreme Gradient Boosting.

To systematically evaluate the predictive efficacy, generalization stability, and overfitting risk of various regression models, this study utilized 1000 iterations of Bootstrap resampling for model performance assessment and bias correction, verifying model reliability across multiple dimensions. Comparative analysis of model performance (Figure 3A, bar chart) revealed significant differences in predictive performance among the models. The RF model achieved the highest median *R* ^2^ (0.96) and the lowest error metrics among all models, with an RMSE of 0.82 μg/mL and an MAE of 0.57 μg/mL; furthermore, the minimal difference between pre‐ and post‐correction metrics indicated stable performance. In contrast, traditional linear models (LR, NB, PR) exhibited mean *R* ^2^ values below 0.40 and significantly higher error metrics. Their performance further declined following correction, indicating apparent overestimation of performance.

Analysis of model stability and bias (Figure 3B, box plot) demonstrated that the three metrics of the RF model had a compact distribution with minimal fluctuation, suggesting high stability in prediction under repeated sampling. Conversely, traditional models displayed wide error distributions and large fluctuations, reflecting poor stability. A comparison of pre‐ and post‐correction results indicated that the RF model possessed minimal bias and controllable overfitting risk, whereas linear models exhibited significant bias and insufficient generalization capability.

Based on these collective findings, the RF model demonstrated significant advantages in predictive accuracy, stability upon repeated validation, and overfitting risk control; consequently, it was selected as the final prediction model for this study.

### 3.4 Evaluation of the RF Model

#### 3.4.1 Assessment of Predictive Performance

**FIGURE 4:**
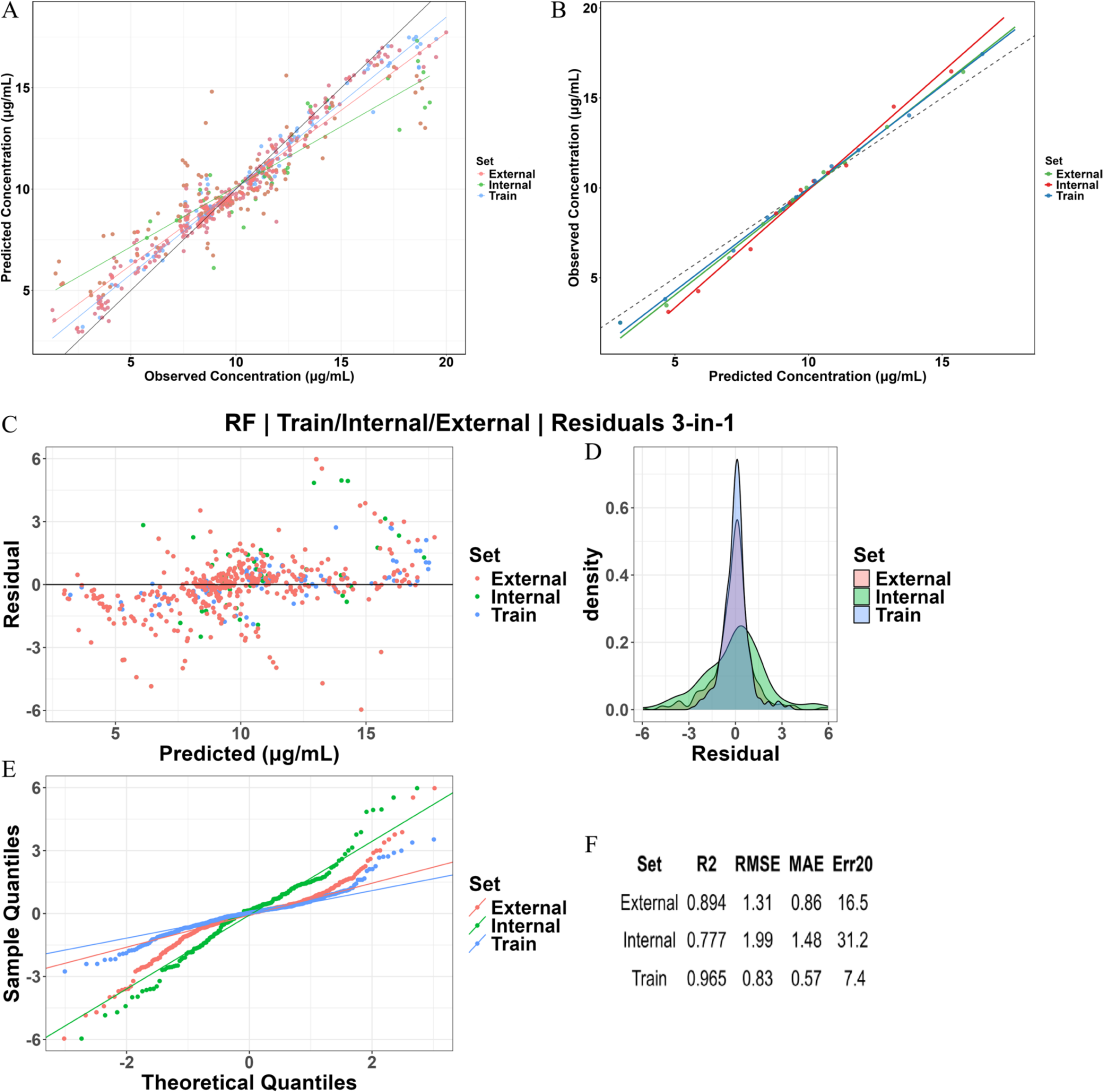
Prediction performance evaluation of the RF model in the training set, internal validation set, and external validation set. (A) Scatter plot of observed versus predicted concentrations; the solid line represents the fitted regression line, while the dashed line indicates the ideal prediction line (*y* = *x*). (B) Calibration curve based on 1000 Bootstrap resamples; the dashed line represents the ideal calibration line (*y* = *x*). (C) Residual distribution plot, showing residuals randomly distributed along the predicted concentration axis. (D) Normal Q‐Q plot of residuals. (E) Density distribution plot of residuals. (F) Summary table of prediction performance metrics across datasets. Evaluation metrics: *R* ^2^ (denotes the coefficient of determination), where a value closer to 1 indicates stronger explanatory power of the model regarding the outcome variable; RMSE (Root Mean Square Error) and MAE (Mean Absolute Error) are error metrics, where lower values indicate higher predictive accuracy.

In this study, the RF regression model was constructed utilizing the training dataset. A comprehensive assessment of the model's predictive capability and generalization performance was conducted using both an internal validation set and an external validation set. The results are presented as follows:

Correlation analysis between observed and predicted values: Scatter plots comparing observed and predicted concentrations across the training, internal validation, and external validation sets (Figure 4A) revealed that all data points were closely distributed around the regression line, exhibiting a significant positive correlation. The training set demonstrated the lowest degree of dispersion. Although the data points in the external and internal validation sets showed some dispersion, their overall trends aligned closely with the line of identity (*y* = *x*), indicating strong concordance between the predicted and observed values.

Calibration curve analysis: Calibration curves constructed via 1000 Bootstrap resamples (Figure 4B) demonstrated that the fitted curves for the training, internal validation, and external validation sets closely adhered to the line of identity (y = x), with no significant systematic bias observed overall. The calibration curve for the training set approximated the ideal state most closely. While the internal and external validation sets exhibited slight upward deviations in the high‐concentration range, the magnitude of deviation was minimal. These findings suggest that the model maintains good calibration across different concentration levels, without significant overfitting or under‐calibration.

Residual analysis and model performance metrics: Residual analysis (Figure 4C) indicated that residuals were randomly distributed on both sides of the zero line across different predicted concentration levels, with no discernible trends or heteroscedasticity. The Q‐Q plot (Figure 4D) further validated the normality assumption of the residuals, confirming that the error distribution satisfies the fundamental prerequisites of regression analysis. Residual density plots (Figure 4E) showed that the residuals for all three datasets approximately followed a normal distribution, with the training set exhibiting the most concentrated distribution, followed by the external validation set, and the internal validation set showing a relatively wider distribution.

Quantitative performance metrics (Figure 4F) demonstrated that the training set achieved a coefficient of determination (*R* ^2^) of 0.97, an RMSE of 0.83 μg/mL, an MAE of 0.57 μg/mL, and an Err20 of 7.4%, indicating excellent goodness of fit. The external validation set yielded an *R* ^2^ of 0.89, an RMSE of 1.31 μg/mL, an MAE of 0.86 μg/mL, and an Err20 of 16.5%. The internal validation set showed an *R* ^2^ of 0.78, an RMSE of 1.99 μg/mL, an MAE of 1.48 μg/mL, and an Err20 of 31.2%. Overall, the model exhibited optimal fitting performance on the training set and maintained robust predictive capability on the external validation set. Although performance on the internal validation set declined slightly, suggesting variability in generalization across different data distributions, the model's performance remained within an acceptable range.

#### 3.4.2 Feature Importance and Decision Logic Analysis of the RF Model

To further elucidate the intrinsic decision mechanism of the random forest model, this study employed the SHAP method for global interpretation and utilized a representative decision tree to visually present feature interaction rules. The results are as follows.

**FIGURE 5:**
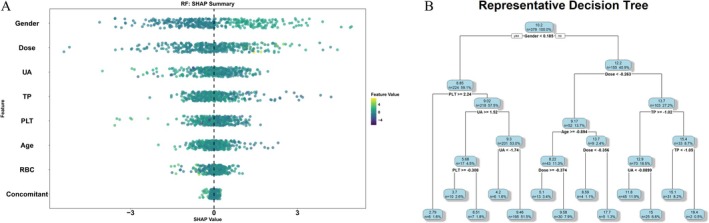
Multidimensional interpretability analysis of the Random Forest model. (A) SHAP feature contribution summary plot. The vertical axis lists features ranked by importance in descending order, while the horizontal axis represents SHAP values. The color of the dots indicates the magnitude of the feature values, and their distribution illustrates the direction and strength of the influence of feature values on the prediction results. (B) Representative decision tree from the Random Forest model (4‐layer depth). Values within nodes represent the average predicted concentration (μg/mL) for that branch, with sample size and proportion annotated below, demonstrating hierarchical interactive decision rules for the features. RBC, red blood cell; PLT, platelet; TP, total protein; UA, uric acid.

The SHAP summary plot (Figure 5A) illustrates the ranking of feature importance: Dose, Gender, TP, and UA were identified as the top four factors influencing drug concentration prediction. Among these, Dose and Gender exhibited the broadest range of SHAP values, indicating the greatest magnitude of impact on prediction outcomes. Dose demonstrated an overall positive contribution trend; samples with high doses (yellow dots) showed significantly positive SHAP values, suggesting that higher administration doses correlate with higher predicted drug concentrations, whereas low‐dose samples exhibited negative contributions, consistent with fundamental pharmacokinetic principles. As a binary feature, Gender displayed a distinct bimodal distribution of SHAP values, indicating systematic differences in the impact of different genders on drug concentration, with specific gender subgroups significantly elevating or lowering the predicted concentration. Regarding laboratory indicators such as TP, UA, and PLT, the SHAP value distributions revealed nonlinear associations with predicted concentrations. For instance, samples with higher TP levels predominantly corresponded to positive SHAP values, suggesting a positive correlation between high TP and elevated drug concentration; conversely, UA and PLT exhibited more complex bidirectional effects, where both low and high levels could influence the predicted concentration in different directions. Features such as Age, RBC, and Concomitant medication showed relatively concentrated SHAP value distributions, contributing less to the prediction results and thus being classified as secondary influencing factors. Notably, Concomitant medication exhibited the narrowest SHAP value distribution, indicating the weakest influence on model decision‐making.

The representative decision tree within the RF (Figure 5B) intuitively presents the hierarchical interaction logic of features. At the root node (first layer), the model initially utilized Gender as the primary splitting feature, dividing samples into two major branches corresponding to different baseline predicted concentrations (8.85 μg/mL vs. 12.2 μg/mL). This directly reflects the predominant impact of gender on drug concentration, aligning with the importance ranking in the SHAP analysis. At the second layer, following gender stratification, the model employed PLT and Dose for secondary splitting to further categorize samples into subgroups, identifying PLT and Dose as key modulating factors within gender subgroups. For example, in the female branch, samples with PLT ≥ 2.24 μg/mL exhibited a significantly higher predicted concentration (9.02 μg/mL) compared to those with PLT < 2.24 (8.85 μg/mL), suggesting that PLT levels play a significant regulatory role in drug concentration for the female population. At the third and fourth layers, the model introduced indicators such as UA, Age, and TP for finer granularity division, forming multiple terminal nodes. For instance, in the high‐dose male subgroup, TP levels further stratified samples into low TP (12.9 μg/mL) and high TP (15.4 μg/mL) branches, and within the high TP subgroup, differences in UA levels further influenced the final predicted concentration. In the low‐dose female subgroup, UA level emerged as a key stratifying factor, where samples with low UA levels showed significantly lower predicted concentrations compared to those with high UA levels.

The decision tree clearly demonstrates the model's hierarchical decision logic: using gender as the primary stratifying factor, combined with the interaction of dose and laboratory indicators, multi‐dimensional drug concentration prediction rules were established, corroborating the feature importance and contribution patterns revealed by SHAP analysis. Synthesizing the SHAP analysis and decision tree results, the random forest model constructed in this study possesses decision logic with clear pharmacokinetic and clinical significance: administration dose is the core driver of drug concentration, while gender, liver function/protein binding‐related indicators (TP), renal function/metabolism‐related indicators (UA), and hematological indicators (PLT) collectively participate in the regulation of drug concentration. This reflects the multi‐factorial regulatory characteristics of the absorption, distribution, metabolism, and excretion processes of the drug in vivo. The above results indicate that the decision rules of the random forest model demonstrate strong consistency with established pharmacokinetic knowledge, providing interpretability support for the clinical application of the model.

## 4 Discussion

Using a cohort of pediatric patients with epilepsy, this study constructed and validated a prediction model for ZNS concentrations based on large‐scale clinical and laboratory data. A rigorous multi‐step feature screening process was employed, encompassing correlation analysis, multicollinearity diagnosis, univariate analysis, Lasso regression, Boruta feature importance assessment, and Bootstrap resampling validation. The RF model was identified as the optimal predictive model, demonstrating robustness and high accuracy in an independent external validation cohort. The study identified key predictive variables influencing ZNS concentration in children, providing a practical predictive tool for individualized dosing that holds significant clinical value for improving the efficacy and safety of pediatric antiepileptic therapy.

### 4.1 Pharmacokinetic Characteristics and Individual Variability of ZNS in Pediatric Epilepsy

ZNS is a broad‐spectrum antiepileptic drug widely used for the treatment of partial and generalized seizures, with increasing application in pediatric patients [16]. Studies indicate that ZNS is characterized by rapid oral absorption, high bioavailability, and a protein binding rate of approximately 40%, with primary excretion via the kidneys in its original form. Its pharmacokinetic profile is influenced by factors such as age, weight, renal function, and concomitant medications, exhibiting significant individual variability [17].

This study strictly adhered to a standardized feature screening protocol. Initially, Spearman correlation analysis and VIF calculations were performed on all variables. The results revealed VIF values exceeding 10 for weight (VIF = 25.06), GFR (VIF = 15.91), and SCr (VIF = 14.54), indicating significant multicollinearity. Consequently, variables with a VIF ≥ 10 were excluded to ensure remaining variables had VIF < 10, thereby preventing feature overlap from compromising subsequent model stability. Given that ZNS is primarily excreted by the kidneys, renal function directly influences drug clearance rate. Furthermore, the strong correlation between body weight and body surface area/glomerular filtration rate in children provides a physiological basis for this collinearity. Direct inclusion of these variables would lead to coefficient inflation, model instability, and increased prediction bias; therefore, indicators with strong collinearity were removed. Multiple studies have confirmed that the development of hepatic and renal function in children is immature, and drug clearance capacity changes significantly with age and weight, leading to marked fluctuations in drug concentrations. The classic study by Wallander et al. [18] noted that weight and renal function are key factors affecting the steady‐state concentration of ZNS. Silva et al. [19] also confirmed in a population pharmacokinetic model established in pediatric patients with refractory epilepsy that weight‐standardized dosing significantly improves the accuracy of concentration prediction. The results of this study are consistent with the aforementioned conclusions; the strong collinearity between weight and SCr/GFR suggests that in the pediatric population, weight can indirectly reflect the developmental level of renal function and is a core physiological indicator affecting ZNS metabolism.

### 4.2 Analysis of Key Factors Influencing ZNS Concentration

In the feature screening process, univariate analysis was conducted on variables that passed the collinearity diagnosis (VIF < 10.00). Variables with statistical significance (*p* < 0.05) were retained, showing that Gender, Dose, Age, RBC, Concomitant medications, TP, UA, and PLT were all significantly correlated with ZNS concentration in children. These eight statistically significant variables were then incorporated into a Lasso regression model. Grid search combined with 10‐fold cross‐validation was used to determine the optimal regularization parameter (*λ* = 0.45). Under the optimal *λ* condition, no variables had their coefficients compressed to zero; thus, all variables were retained as candidate features. Finally, the candidate variables retained by Lasso regression were subjected to feature importance evaluation using the Boruta algorithm and stability verification via 500 Bootstrap resamples, cross‐validated with the Random Forest feature importance ranking. This process definitively identified Gender, Dose, Age, RBC, Concomitant medications, TP, UA, and PLT as important predictive variables for ZNS concentration, which were used for the construction of the subsequent prediction model. These results are partially consistent with existing research conclusions, although certain discrepancies exist due to differences in study populations, included indicators, and analytical methods.

Gender and age, as basic physiological indicators, affect the concentration and in vivo distribution of ZNS, but conclusions from different studies vary. In pediatric research, analysis of the relationship between gender and clearance rate showed no statistically significant difference between male and female subjects, suggesting that during childhood development, gender may not be a primary factor influencing drug metabolism [18]. This result differs from the present study, which found gender to be a key factor influencing ZNSconcentration (SHAP analysis showed its importance was second only to Dose). In a study involving healthy Chinese adult volunteers, pharmacokinetic data after a single dose showed significant differences in ZNS concentrations between male and female subjects [20]. This discrepancy may stem from the regulatory effects of sex hormones on drug‐metabolizing enzymes, changes in volume of distribution caused by body fat percentage differences, and gender differences in gastrointestinal absorption rates.

Multiple studies have demonstrated that dosing regimen is a critical determinant of ZNS concentration. Kimura et al. [21] found a significant positive correlation between the weight‐adjusted daily dose of ZNS and steady‐state concentration. Silva et al. [19] also confirmed that ZNS trough concentrations increase with dosage. Notably, for the same total daily dose, a twice‐daily regimen results in higher trough concentrations than a once‐daily regimen. For instance, median trough concentrations for 100.00 and 200.00 mg once‐daily dosing were both below the therapeutic window (4.84 and 9.40 mg/L, respectively), whereas the 100.00 mg twice‐daily regimen remained at a sub‐therapeutic level. SHAP analysis in this study showed that Dose had the greatest impact on ZNS concentration, exhibiting an overall positive contribution trend. High‐dose samples had significantly positive SHAP values, indicating that higher doses predict higher drug concentrations, which aligns with basic pharmacokinetic principles. This finding has significant pharmacological implications: ZNS clearance in children is positively correlated with weight, and weight‐adjusted dosing can eliminate the influence of body size differences. Miro et al. [22] pointed out that dosing regimen is the strongest predictor of ZNS exposure. This study further confirms that in nonlinear machine learning models, dosing regimen remains the primary influencing factor, highlighting its central role in pediatric ZNS administration.

This study confirms that UA is a key variable influencing the concentration of ZNS. SHAP analysis revealed that its importance ranks third, exhibiting a nonlinear association with ZNS concentration; both low and high levels of UA may exert effects in different directions on the predicted concentration. Although current research on the relationship between UA and ZNS is limited, existing evidence indicates that patients' pathological conditions and renal function significantly affect the efficiency of drug metabolism and excretion. As an endogenous antioxidant marker, UA can reflect renal function and oxidative stress status [23]. It is worth noting that both ZNS and UA are secreted and excreted via the proximal renal tubules, suggesting a competitive excretion mechanism between the two. When renal function is impaired, the clearance rate and protein binding rate of ZNS may be altered, leading to disturbances in the in vivo pharmacokinetic environment [24]. Therefore, elevated UA levels may reduce the excretion efficiency of ZNS, subsequently leading to increased plasma concentrations. Literature has reported a case of a dog with idiopathic epilepsy that developed distal renal tubular acidosis and lethargy after ZNS administration, manifested as normal anion gap metabolic acidosis, hypokalemia, hyperchloremia, and alkaline urine; this case reveals a potential mechanism by which ZNS may interfere with renal tubular function [25]. Based on the above findings, it is recommended to consider UA as a simple reference indicator for ZNS dose adjustment in clinical practice; for pediatric patients with hyperuricemia, the ZNS dose should be appropriately reduced and medication monitoring strengthened.

Drugs exist in the blood in two forms: bound and free, with the free form possessing pharmacological activity, being metabolized and cleared, and potentially inducing toxic reactions. When liver function impairment leads to decreased total protein levels, the carrier capacity for drug binding becomes insufficient, resulting in elevated free drug concentrations and thereby increasing the risk of adverse reactions [26]. The protein binding rate of ZNS is approximately 40.00%; an increase in total protein levels will decrease the free drug concentration, thereby affecting the apparent clearance rate and plasma concentration. The first reported case of ZNS‐associated acute‐on‐chronic liver failure in the literature showed that the patient developed jaundice and mild encephalopathy after 13 days of medication, and laboratory examinations indicated significant liver function abnormalities, suggesting that patients with hepatic insufficiency may experience ZNS metabolic disorders and require close monitoring of liver function and drug concentration [27]. This study confirms a nonlinear association between TP and ZNS concentration. SHAP analysis showed that samples with higher TP levels mostly corresponded to positive SHAP values, suggesting that high TP may be associated with elevated drug concentrations, which is consistent with drug‐protein binding theory. Therefore, in antiepileptic drug therapy, solely monitoring drug concentration is insufficient to comprehensively assess pharmacological effects and toxicity risks, especially for patients with conditions such as liver function impairment or malnutrition that may lead to hypoproteinemia. It is recommended to simultaneously monitor liver function indicators as well as plasma total protein and albumin levels to accurately assess the drug metabolism status, avoiding adverse reactions caused by excessively high free drug concentrations or insufficient dosage.

The results of this study indicate that RBC is an important factor influencing ZNS concentration; however, SHAP analysis showed that its contribution to the prediction results is relatively small, classifying it as a secondary influencing factor. Meanwhile, SHAP analysis ranked gender among the top four key variables for model prediction (second only to dose), indicating a significant impact on ZNS concentration. This finding is consistent with the population pharmacokinetics study results by Qiu et al. [28] in healthy Chinese volunteers, confirming significant gender differences in ZNS concentration and that gender affects intercompartmental clearance. However, Silva et al. [19] reached the opposite conclusion, suggesting that gender does not affect ZNS clearance. This discrepancy may stem from differences in study subjects: Silva et al. focused on adult patients, whereas this study focused on pediatric patients. Physiological differences between genders during childhood development (such as body weight, fat distribution, and maturity of liver and kidney function) are more significant, potentially leading to gender‐dependent differences in drug metabolism and distribution. Leppik et al. [29] indicated that while the binding rate of ZNS to plasma proteins is not high, its affinity for RBC is 8 times that of plasma proteins, and significant drug concentration phenomena can be observed in human RBC. Since RBC counts are typically higher in males than in females, this may lead to stronger drug‐RBC binding in male patients, followed by slow drug release and elimination. This mechanism may explain the phenomenon of lower peak concentrations and slower elimination rates in male patients, while also highlighting the unique impact of gender on the pharmacokinetics of ZNS in children. El‐Khateeb et al. [30] also confirmed that RBCs possess a significant reservoir effect for highly erythrophilic drugs (such as tacrolimus); through drug binding and slow release, RBCs can stabilize plasma concentrations, prolong drug half‐life, and reduce peak concentration fluctuations, thereby influencing pharmacokinetic characteristics, which is consistent with the results of this study. Therefore, it is recommended to comprehensively consider patient gender and RBC levels when formulating ZNS dosing regimens in clinical practice to achieve individualized dosing and improve the rate of treatment target attainment.

In variable screening, although “concomitant medication” was significantly correlated with ZNS concentration, SHAP analysis showed that its SHAP value distribution was the narrowest, and its contribution to the prediction results was the smallest; thus, it was not listed among the top four key predictive variables. Previous studies have shown that enzyme‐inducing antiepileptic drugs (such as phenytoin and carbamazepine) can significantly reduce ZNS concentration, whereas drugs like sodium valproate, levetiracetam, and oxcarbazepine have minor effects [31, 32]. The concomitant drugs in this study were primarily sodium valproate, levetiracetam, oxcarbazepine, and lamotrigine; therefore, concomitant medication was not identified as a key variable. However, it should be noted that combination therapy remains an important factor influencing ZNS pharmacokinetics. This study did not comprehensively evaluate the effects of different concomitant medications; future studies need to expand the sample size and increase drug combinations to systematically analyze the impact of drug interactions on ZNS concentration.

### 4.3 Comparative Analysis of Machine Learning Model Prediction Efficacy

Existing studies indicate that domestic and international research on ZNS pharmacokinetics has mainly focused on the field of population pharmacokinetics. Hashimoto et al. [33] analyzed ZNS pharmacokinetic characteristics using therapeutic drug monitoring data from 68 epilepsy patients; Okada et al. [34] evaluated the effects of CYP2C19 and CYP3A5 gene polymorphisms on ZNS clearance; Qiu et al. [28] established a ZNS population pharmacokinetics model for healthy volunteers; Silva et al. [19] constructed the first population pharmacokinetics model for adult patients with refractory epilepsy in Europe. Traditional population pharmacokinetics models are based on preset mathematical structures (with compartment models as the core) and possess clear physiological interpretability. Their core advantage lies not only in predicting trough plasma concentrations but also in accurately predicting plasma concentrations at any time point. Simultaneously, they can calculate key pharmacokinetic parameters such as area under the curve, time to peak, and half‐life, clarifying the entire process of absorption, distribution, metabolism, and excretion of the drug in the body, which is irreplaceable by machine learning models [19]. In contrast, the core advantage of machine learning models lies in not requiring preset strict mathematical assumptions and compartment structures; they can automatically mine complex nonlinear relationships between clinical indicators and ZNS concentration, as well as interactions between variables. They exhibit stronger adaptability to high‐dimensional clinical data with multicollinearity and offer higher accuracy and greater convenience in rapid prospective prediction of plasma concentrations [35, 36]. They can quickly provide preliminary references for clinical dose adjustment, compensating for the deficiencies of compartment models in rapid prediction and complex data processing; however, they cannot fully reveal the dynamic laws of the in vivo drug process, nor can they easily provide core pharmacokinetic parameters beyond concentration prediction.

It is crucial to clarify that the machine learning algorithms constructed in this study, such as RF, are not intended to replace traditional compartmental pharmacokinetic (PK) models. Instead, they represent a complementary and synergistic approach aimed at providing comprehensive support for the individualized dosing of ZNS in children. Currently, research applying machine learning to ZNS is limited; existing predictive models for pediatric antiepileptic drugs predominantly focus on sodium valproate and levetiracetam, with a specific predictive model for ZNS in children yet to be established [37]. Furthermore, in the construction of traditional compartmental PK models, covariate screening often relies on empirical judgment, which is susceptible to covariate omission and multicollinearity, thereby compromising model accuracy. This study employed a multi‐step feature screening strategy—encompassing correlation analysis, collinearity diagnostics, univariate analysis, Lasso regression, Boruta feature importance assessment, and Bootstrap stability verification‐to effectively address these limitations. This approach leverages the objectivity of machine learning in variable selection to automatically identify core covariates most relevant to ZNS concentration without significant collinearity (e.g., Dose, Gender, TP, UA). These screened covariates were subsequently applied to the construction of the compartmental PK model, minimizing interference from irrelevant variables, optimizing model structure, and enhancing the fitting accuracy of the compartmental model regarding in vivo drug processes and parameter estimation [13]. This paradigm of “machine learning‐assisted covariate screening combined with compartmental modeling for revealing dynamic drug processes” achieves complementary advantages: the machine learning model facilitates rapid concentration prediction and objective covariate screening, while the compartmental model offers strong physiological interpretability and comprehensive parameters. This synergy resolves the scientific limitations of covariate screening in compartmental models and compensates for the inability of machine learning to elucidate dynamic in vivoprocesses, thereby further enhancing the scientific rigor and precision of individualized dosing regimens.

Experimental results demonstrate the significant advantages of the machine learning model (RF). The RF model exhibited the best performance among 12 comparative models (LR, NB, PR, DT, RF, SVR, GBDT, KNN, ANN, MLP, XGBoost, LightGBM). In 1000 Bootstrap resampling validations, it achieved the highest median *R* ^2^ (0.96) and the lowest error metrics (RMSE: 0.82 μg/mL; MAE: 0.57 μg/mL), with minimal difference between pre‐ and post‐correction metrics, indicating stable performance. These findings align with existing research on antiepileptic drugs, which indicates that for four common antiepileptic drugs (carbamazepine, phenobarbital, phenytoin, and valproic acid), the predictive performance of 10 artificial intelligence models generally surpasses that of traditional population pharmacokinetic models, with ensemble learning methods (e.g., RF, AdaBoost, and XGBoost) performing particularly well [38]. This superiority is primarily attributed to the ability of machine learning models to effectively capture complex nonlinear relationships between ZNS concentrations and influencing factors. Through bootstrap sampling and random feature selection, the RF model reduces overfitting risk and enhances prediction stability. Conversely, traditional linear models (LR, NB, PR) yielded mean *R* ^2^ values below 0.4 with significantly higher error metrics, and their performance declined further after correction, indicating an inability to handle such complex associations. Meanwhile, gradient boosting models (GBDT, XGBoost, LightGBM) performed second only to RF but with lower prediction accuracy, further validating the advantage of the RF model in this study and underscoring the application value of machine learning in blood concentration prediction.

The RF regression model constructed in this study based on the training set (375 cases) was comprehensively evaluated using an internal validation set (157 cases) and an external validation set (436 cases). It demonstrated excellent predictive efficacy and robust generalization capability, with performance metrics highly consistent with the model's characteristics, the data validation design, and clinical requirements. Correlation analysis between observed and predicted values revealed that sample points across all three datasets closely adhered to the fitted line, with overall trends highly consistent with the ideal prediction line. The training set exhibited the lowest dispersion, a result closely related to the algorithmic characteristics of the RF model. By aggregating predictions from multiple decision trees, the RF model effectively mitigates the overfitting risk associated with single decision trees and fully captures complex associations between features and ZNS concentration in the training set. This makes it particularly suitable for prediction scenarios involving multi‐variable interactions (8 features including Gender, Dose, TP) in this study, consistent with findings from previous studies on similar drugs [37]. The external and internal validation sets showed some dispersion, likely due to individual heterogeneity. Although the internal validation set originated from the same modeling cohort as the training set, unincorporated individual differences (e.g., potential genetic polymorphisms, dietary influences) may exist. The external validation set, derived from an independent cohort, may reflect clinical practice differences across centers, causing minor deviations between predicted and observed values. However, the overall consistency remained good, indicating the model's ability to capture core determinants of ZNS concentration. In calibration curve analysis, the fitted curves for all three datasets closely approximated the ideal calibration line, with the training set showing optimal calibration. The internal and external validation sets exhibited only slight upward deviation in the high‐concentration range, suggesting no significant systematic bias and controllable risks of overfitting and under‐calibration. These results validate the rationality of the RF model's parameter settings; appropriate tree quantity and depth prevented both underfitting (failure to capture complex associations) and overfitting (poor generalization). The slight upward deviation in the high‐concentration range may be related to the pharmacokinetic characteristics of ZNS; at certain concentration levels, metabolic enzymes (e.g., hepatic cytochrome P450 system) may become saturated, leading to non‐linear kinetics where clearance rate decreases. The model's slight limitation in capturing this non‐linearity represents a key focus for future optimization. Residual analysis further confirmed model reliability: residuals were randomly distributed around the zero line with no obvious trends or heteroscedasticity, approximating a normal distribution. This aligns with regression analysis assumptions, indicating reasonable error distribution and absence of systematic error sources. The training set residuals were most concentrated, followed by the external validation set, with the internal validation set being relatively more dispersed. This trend is consistent with quantitative performance metrics: the training set achieved an *R* ^2^ of 0.97, RMSE of 0.83 μg/mL, and MAE of 0.57 μg/mL, indicating excellent fit; the external validation set maintained an *R* ^2^ of 0.89, with RMSE of 1.31 μg/mL and MAE of 0.86 μg/mL, showing a moderate increase in error but maintaining good predictive accuracy in a new population; the internal validation set showed an *R* ^2^ of 0.78, with RMSE of 1.99 μg/mL and MAE of 1.48 μg/mL. While error metrics increased notably, suggesting some generalization variance within subsets of the same population, the *R* ^2^ > 0.70 and Err20 = 31.20% remain within acceptable ranges for clinical concentration prediction models. Compared to similar studies, the RF model constructed here demonstrates superior predictive performance. Previous studies on ZNS concentration prediction in epileptic children using traditional linear regression models typically reported *R* ^2^ values between 0.50 and 0.70 with higher error metrics, primarily because linear models cannot capture nonlinear associations between features and concentration. In contrast, the RF model effectively fits complex nonlinear relationships under multi‐variable interactions, making it particularly suitable for pediatric populations where physiological immaturity leads to significant individual differences in weight, age, and hepatorenal function affecting drug metabolism, along with potential interactions among these indicators. The ensemble learning characteristics of the RF model are well‐suited to this complex scenario. Furthermore, the three‐layer validation design (“training set fitting – internal validation set optimization – external validation set validation”) adopted in this study provides a more objective reflection of generalization ability than single validation methods, avoiding performance overestimation and enhancing the reliability of the conclusions.

Further analysis was conducted on the clinical significance of the model's absolute error by integrating error characteristics with clinical application scenarios. The optimal RF model demonstrated a maximum MAE of 1.48 μg/mL and a maximum RMSE of 1.99 μg/mL across datasets, indicating a controllable overall error level. Given that the effective therapeutic concentration window for ZNS in pediatric epilepsy treatment ranges from 20.00 to 40.00 μg/mL, the observed MAE is substantially lower than the width of the therapeutic window. This margin of error is insufficient to cause misinterpretation of concentration levels, thereby effectively meeting clinical requirements for routine dose adjustment, concentration prediction, and preliminary efficacy assessment. Analysis of regression results revealed a trend where the model slightly overestimates low concentrations and underestimates high concentrations; this pattern is an inherent characteristic of tree‐based algorithms. The RF algorithm optimizes for the minimization of the global loss function, resulting in the shrinkage of predicted values toward the overall mean. This inherent bias does not constitute a modeling defect and may offer clinical benefits: slight overestimation in the low‐concentration range mitigates the risk of blind dose escalation due to underestimation of effective concentrations, while slight underestimation in the high‐concentration range prevents inappropriate dose reduction due to overestimation, thereby collectively enhancing clinical medication safety. However, the application limitations of the model must be objectively acknowledged. In scenarios requiring precise dose titration for high‐dose regimens, or for special populations with hepatic or renal impairment and complex polypharmacy, the minor prediction errors may limit the model's utility, indicating that it cannot completely replace real‐time therapeutic drug monitoring. Nevertheless, for the more prevalent clinical scenarios involving routine medication guidance and preliminary screening for typical pediatric epilepsy patients, the error level is within a clinically acceptable range, demonstrating the model's practical utility. Future studies should focus on expanding sample sizes for special populations, optimizing loss function weights for extreme concentration intervals, and incorporating potential metabolic regulatory features such as genetic polymorphisms to further reduce prediction errors in extreme ranges and enhance the model's adaptability and accuracy in refined, individualized medication scenarios.

The novelty of this study is primarily characterized by three key advancements. First, a rigorous multi‐step feature screening strategy was implemented, integrating correlation analysis, collinearity diagnosis (excluding variables with VIF ≥ 10.00), univariate analysis (retaining variables with *p* < 0.05), Lasso regression (with the optimal *λ* determined by 10‐fold cross‐validation), Boruta feature importance assessment, and 500‐iteration Bootstrap validation. This approach leveraged the strengths of machine learning in variable selection, effectively mitigated multicollinearity, and ensured the scientific validity and accuracy of identifying critical predictors for ZNS concentration. Furthermore, it provided a robust methodology for covariate screening in compartmental pharmacokinetic models. Second, a comparative analysis of 12 distinct prediction models identified Random Forest as the optimal algorithm. This selection overcame the limitations inherent in traditional linear models, highlighting the superior accuracy and generalization capabilities of machine learning in predicting concentrations, thereby facilitating rapid clinical forecasting. Third, the model's generalizability was rigorously confirmed through both internal and external validation. The integration of SHAP analysis enhanced model interpretability, while representative decision trees elucidated feature interaction rules. Consequently, the model not only possesses predictive utility but also clarifies the regulatory roles and hierarchical interactions of key variables, offering targeted references for clinical individualized dosing and covariate optimization in compartmental pharmacokinetic models, thus achieving a synergistic complementarity between the two modeling approaches.

It is important to note that the prediction model was constructed using baseline clinical and laboratory data obtained after ZNS dose stabilization but prior to TDM sampling, rather than synchronous indicators measured on the day of sampling. This design fundamentally circumvents the critique that “direct measurement of concentration is more reliable,” thereby clarifying the model's incremental clinical value. The core utility of the model is not to replace concentration testing or compartmental pharmacokinetic models, but to leverage the rapid prediction capabilities of machine learning. It enables the prospective prediction of steady‐state ZNS concentrations in pediatric patients before TDM results are available, assisting in the early optimization of dosing regimens, reducing the burden of repeated sampling, and shortening the dose titration cycle. Simultaneously, the core covariates identified can assist in optimizing compartmental pharmacokinetic models, enhancing their ability to elucidate in vivo drug processes. This is particularly valuable in clinical scenarios involving pediatric patients, who exhibit rapid growth and significant inter‐individual pharmacokinetic variability, providing timely and reliable references for individualized dosing. Furthermore, compartmental pharmacokinetic models can build upon the predictions of the machine learning model to provide dynamic in vivo parameters, offering comprehensive theoretical support for dose adjustments.

Compared to traditional population pharmacokinetic models with covariate structures, the machine learning model constructed in this study demonstrates distinct advantages and complementary value. Population pharmacokinetic models rely on preset compartmental structures and fixed covariate relationships, presenting limitations in handling complex non‐linear associations and multicollinear clinical indicators. Additionally, individualized prediction typically requires Bayesian prior information and subsequent concentration feedback updates, rendering the process relatively cumbersome and time‐consuming. In contrast, the Random Forest model employed here does not require a pre‐defined mathematical structure; it automatically mines non‐linear relationships and interactions among variables, exhibiting stronger adaptability to high‐dimensional, collinear clinical data. It offers rapid prediction and operational convenience without waiting for TDM results or Bayesian updates, enabling rapid optimization of regimens during the early titration phase. The covariates screened effectively compensate for the deficiencies in covariate selection within compartmental models. Meanwhile, compartmental pharmacokinetic models can further reveal dynamic in vivo drug changes based on the machine learning predictions, providing key parameters such as AUC and time to peak, thereby offering comprehensive theoretical support and compensating for the machine learning model's inability to resolve in vivo drug processes. The combination of SHAP analysis quantifies the direction and magnitude of each factor's influence on concentration, while decision trees clarify feature interaction rules, further enhancing model interpretability and clinical operability, and providing references for covariate weight allocation in compartmental models. Therefore, this machine learning model does not replace the traditional population pharmacokinetics framework but serves as a vital supplement in rapid individualized prediction, complex relationship fitting, and covariate screening. It acts synergistically with compartmental pharmacokinetic models, making it highly suitable for real‐world clinical scenarios of individualized ZNS dosing in pediatric patients with epilepsy.

However, this study has certain limitations. First, all subjects were recruited from a single region, which may introduce selection bias. Future studies should expand the sample source to include pediatric epilepsy patients from different regions, age groups, and disease conditions to enhance the model's representativeness and ensure the screened covariates are applicable to compartmental pharmacokinetic modeling across diverse populations. Second, genetic polymorphism indicators were not included; however, the genetic polymorphism of drug‐metabolizing enzymes is a significant factor influencing the concentration of antiepileptic drugs. Future research should integrate genetic testing data to optimize model structure and prediction accuracy, while incorporating genetic polymorphism indicators alongside screened core covariates into compartmental pharmacokinetic models to improve physiological interpretability [39]. Finally, the retrospective nature of this study implies potential missing information or measurement errors during data collection. Future prospective studies are warranted to minimize the influence of confounding factors, further validate the clinical utility of the model, and assess the efficacy of the “machine learning‐assisted covariate screening plus compartmental model” approach, fully realizing the synergistic advantages of both modeling types.

## 5 Conclusion

This study successfully constructed and validated a Random Forest‐based prediction model for ZNS concentration in children. Constructed using baseline clinical and laboratory indicators obtained after dose stabilization but prior to TDM sampling, the model enables prospective prediction of steady‐state concentrations before actual measurements are available. This effectively avoids the issue of “insufficient additive value due to reliance on synchronous sampling data” and highlights the advantages of machine learning: high accuracy, robust stability, strong generalization, and rapid prediction. Combined with SHAP analysis and representative decision trees, the regulatory roles and interaction rules of key variables were further elucidated, enhancing the model's interpretability.

Dose, Gender, UA, and TP were identified as core variables influencing ZNS concentration (top four in SHAP analysis), while Age, RBC, Concomitant medications, and PLT were secondary influencing variables. Based on routine clinical indicators requiring no specialized testing, this model can be directly applied to the optimization of individualized ZNS dosing in children. It assists clinicians in predicting concentrations prior to TDM implementation, reducing the need for repeated sampling monitoring and shortening the dose titration cycle. Furthermore, the core covariates screened can assist in optimizing traditional compartmental pharmacokinetic models, resolving issues regarding unscientific covariate selection and susceptibility to collinearity interference. This fosters a complementary advantage: the machine learning model achieves rapid concentration prediction and objective covariate screening, while the compartmental model reveals dynamic in vivo processes and provides comprehensive pharmacokinetic parameters. Together, they provide robust support for individualized ZNS dosing in children. This study not only highlights the application value of machine learning in predicting pediatric ZNS concentrations but also leverages the physiological analytical strengths of compartmental pharmacokinetic models, holding significant clinical value and broad application prospects for improving the safety, efficacy, and precision of pediatric ZNS therapy.

## Author Contributions

Li Fashuang, Li Na, Ma Mingbiao, and Li Huiying designed the study. Li Fashuang, Li Huiying, and Ma Mingbiao conducted data collection; Li Fashuang, Li Na, Li Huiying, and Ren Danyang completed the data analysis. Li Fashuang and Tu Caixia wrote the manuscript. Li Fashuang, Ma Mingbiao, Li Na, Li Linbo, and Zhang Lilin supervised the project. All authors contributed to critically reviewing the article and approved the submitted version.

## Funding

This study was supported by the Science and Technology Plan Project of Yunnan Province (202301AY070001‐280, 2024YNLCYXZX0440); Special Program for Training High‐Level Health Professionals of Yunnan Province (H‐2024061); Science and Technology Achievement Transformation Project of Yunnan Province (YX‐2023‐02); “Hundred Talents” Project for Health Science and Technology Talents Training of Kunming (2024‐SW9(Lead)‐16).

## Ethics Statement

The study protocol received ethical approval from the Institutional Review Board of the Affiliated Children's Hospital of Kunming Medical University (No. 2026‐05‐057‐K01).

## Conflicts of Interest

The authors declare no conflicts of interest.

## Data Availability

The data that support the findings of this study are available on request from the corresponding author. The data are not publicly available due to privacy or ethical restrictions.
